# Schoolteachers' Resilience Does but Self-Efficacy Does Not Mediate the Influence of Stress and Anxiety Due to the COVID-19 Pandemic on Depression and Subjective Well-Being

**DOI:** 10.3389/fpsyt.2021.756195

**Published:** 2021-10-06

**Authors:** Inn-Kyu Cho, Jihoon Lee, Kyumin Kim, Joohee Lee, Sangha Lee, Soyoung Yoo, Sooyeon Suh, Seockhoon Chung

**Affiliations:** ^1^Department of Psychiatry, Asan Medical Center, University of Ulsan College of Medicine, Seoul, South Korea; ^2^Department of Convergence Medicine, University of Ulsan College of Medicine, Seoul, South Korea; ^3^Department of Psychiatry, Ajou University School of Medicine, Suwon, South Korea; ^4^Department of Psychology, Sungshin Women's University, Seoul, South Korea

**Keywords:** stress, psychology, teacher, COVID-19, anxiety

## Abstract

**Objectives:** In the current coronavirus disease (COVID-19) pandemic, schoolteachers experience stress from addressing students or performing school tasks that may result in burnout. This study aimed to observe whether teachers' stress and anxiety due to the pandemic can influence their depression or psychological well-being and examine whether their resilience or self-efficacy mediates this association.

**Methods:** During March 4–15, 2021, 400 teachers participated and responded voluntarily to an online survey that included the Stress and Anxiety to Viral Epidemics-9 items (SAVE-9), the Teacher-Efficacy Scale, the Brief Resilience Scale (BRS), the WHO-5 Well-Being Index, and the Patients Health Questionnaire-9 items.

**Results:** High psychological well-being of teachers in COVID-19 pandemic era was expected by a low SAVE-9 score (aOR = 0.95; 95% CI, 0.91–0.99), a high level of self-efficacy (aOR = 1.03; 95% CI, 1.01–1.06), and a high BRS score (aOR = 1.18; 95% CI, 1.10–1.27). Moreover, teachers' resilience mediated the effects of stress and anxiety from the COVID-19 pandemic on their subjective well-being or depression.

**Conclusions:** Schoolteachers' subjective well-being and depression were influenced by high levels of stress and anxiety of the viral epidemic, and their resilience mediated this relationship in this COVID-19 pandemic era.

## Introduction

In December 2019, a new type of viral pneumonia was detected in Wuhan City, China, which announced the beginning of a novel pandemic 10 years after the H1N1 influenza pandemic in 2009 (1). The outbreak in South Korea started in February 2020, when a person living in Daegu was diagnosed with coronavirus disease (COVID-19), and hundreds of people were infected by the virus in the subsequent days (2). As of August 19, 2021, 19 months after the virus was first identified in Korea, the total number of confirmed COVID-19 cases in South Korea was 230,808^1^. As the COVID-19 pandemic worsened, many changes occurred in the school learning environment. Schools or educational institutions were temporarily closed, and the educational systems rapidly adopted online teaching (3). In 2020, the schools' opening day was delayed from March 1 to mid-May in Korea (4), and schoolteachers faced grave problems even after the schools reopened. Teachers play an active role in preventing the spread of viruses throughout schools. They must check or rate students' temperatures or suspected symptoms and ensure that the students are wearing their masks.

### Depression and Psychological Well-Being of Schoolteachers During COVID-19 Pandemic

There are few studies on the mental health condition of schoolteachers in the COVID-19 pandemic era. According to a cross-sectional survey conducted in China, depression was common among teachers during the COVID-19 outbreak (5). Another study comparing depression in early educators in the state of Virginia, US, before and during the pandemic also showed a significant increase in depression rates, especially for school-based teachers^2^. Furthermore, a cross-sectional study of 139 professional teachers in the Philippines showed that COVID-19 moderately impacted their quality of life (QOL) (6).

Although no study has examined the relationship among anxiety, depression, and the psychological well-being of schoolteachers in this pandemic, some demonstrated it in several other populations. A survey in Oman concluded that physicians' overall well-being was strongly influenced by both stress and anxiety in the pandemic (7). A study in Turkey examining the mental well-being of healthcare professionals and non-healthcare professionals in the pandemic era showed that psychological well-being had a significant negative correlation with depression and anxiety (8). Another study conducted in Pakistan reported that both COVID-related anxiety and perceived stress are negatively associated with psychological well-being among adult populations (9).

### Mediating Role of Resilience and Self-Efficacy

Resilience can be briefly defined as the ability to maintain one's mental health in a stressful situation (10). For teachers, numerous situations can lead to minor or intense stress. New stress factors for teachers emerged during the COVID era, including inevitable changes in the teaching environment, such as socially distanced classrooms or hybrid teaching and increased uncertainty (11). In the case of teachers, resilience demonstrates their capability of adapting positively while managing such situations. Multiple risk and protective factors affect teachers' resilience. Negative self-belief or confidence is one risk factor, while sense of humor and patience are protective factors (12). Teachers' resilience is known to influence their job satisfaction, eventually leading to professional growth (13). According to descriptive research conducted in Turkey, the higher the teachers' resilience levels, the lower the burnout rates. Moreover, teachers' resilience levels were significantly correlated with their organizational commitment, job satisfaction, and perception of the organizational climate (14).

Self-efficacy, a popular concept in social cognitive theory for decades, refers to an individual's perception or belief of their ability to perform tasks in their workplace (15). Furthermore, teacher efficacy is the teacher's belief in their capability to provide an adequate learning environment and properly instruct and communicate with their students. Previous research shows the effects of self-efficacy on various issues that teachers may encounter and the effectiveness of their teaching and classroom management (16, 17). Regarding the changes in teaching environment, the importance of teacher efficacy is emphasized in the pandemic. For instance, teachers' confidence plays a major role in promoting successful online teaching (18). From the teachers' viewpoint, efficacy is proven to reciprocally influence burnout, adaptability, and job satisfaction, which are important risk factors for mental problems (19).

In a pandemic, schoolteachers' anxiety responses may be associated with depression and their psychological well-being; their resilience or self-efficacy can mediate these associations. Thus, in this study, we hypothesized that schoolteachers' stress and anxiety response to the viral epidemic would directly explain variations in their psychological well-being, depression, resilience, and self-efficacy. We also expected that resilience or self-efficacy of schoolteachers would directly contribute to their depression or psychological well-being. Furthermore, we expected that resilience or self-efficacy would play a mediating role in the relationship between stress and anxiety to the viral epidemic and depression or psychological well-being.

## Methods

### Participants and Procedure

This survey was conducted via an online platform of a professional survey company (embrain.com). The target population was schoolteachers working at the elementary, middle, and high school levels in South Korea. The survey was conducted March 4–15, 2021. The participants responded to the survey voluntarily. The study protocol was approved by the Institutional Review Board of Asan Medical Center (2021-0098), which waived the need for written informed consent.

The survey included questions on participant age, sex, marital status, workplace, and years of employment. It also captured history of quarantine for COVID-19 and experience caring for children who have had viral illness. Past psychiatric history and current psychiatric symptoms were also included. The survey was developed in Korean and followed the Checklist for Reporting Results of Internet e-Surveys (CHERRIES) guidelines (20). After its development, the usability and technical functionality of the electronic questionnaire set by the survey company was tested by an investigator (SC) before its implementation.

The sample size estimation was 30 participants per cell (21). We allocated 30–40 samples for 10 cells—biological sex (two groups) by age (five groups), and all 400 participants were enrolled from the 14 million general population panels of the survey company. The panelists voluntarily provided their information and participated in the survey. The company sent emails to 4,000 schoolteacher panelists for study enrollment and collected all 400 participants' responses among 854 panelists who accessed the survey system. This represents 9.23% of all registered schoolteachers (433,284) in South Korea^3^. The company collected the participants' responses and delivered the collected data to the investigators after excluding all identifiable private information.

### Symptom Assessment

#### Schoolteachers' Version of Stress and Anxiety to Viral Epidemics-9 Items

The Stress and Anxiety to Viral Epidemics-9 (SAVE-9) scale was originally developed for the assessment of work-related stress and anxiety responses of healthcare workers (22). In this study, we used the teacher version of SAVE-9 scale [(23); www.save-viralepidemic.net]. The respondents answered each item on a 5-point Likert scale ranging from 0 (never) to 4 (always). Since the SAVE-9 scale was originally developed in Korean, we used its original version.

#### Patient Health Questionnaire-9 Items

The Patient Health Questionnaire-9 items (PHQ-9), a self-administered nine-item questionnaire, assesses depression symptoms. Each item is scored on a four-point Likert scale from 0 (not at all) to 3 (nearly every day). Scores can range from 0 to 27, with higher scores reflecting greater symptom severity (0–4 = minimal depression; 5–9 = mild depression; 10–14 = moderate depression; 15–19 = moderately severe depression; and ≥20 = severe depression) (24). In this study, we applied the Korean version of the PHQ-9 scale (25).

#### Teachers-Efficacy Scale

To assess teachers' efficacy, we used the Teacher-Efficacy Scale validated from the original scale (26). This scale has 25 items categorized into three subscales: seven items of self-confidence, 11 items of self-regulatory efficacy, and seven items of task difficulty preference. The respondents answered each question from 1 (never) to 6 (strongly agree), and 10 items were reverse-scored. This scale was originally validated in Korean (26).

#### Brief Resilience Scale

The Brief Resilience Scale (BRS) was developed to measure one's resilience, that is, one's capacity to recover quickly from difficult situations (27). The respondents answered six items ranging from 1 to 5 for a total score of 6–30, and three items were reverse-scored. A higher score implies a high level of resilience. In this study, the Korean version was used (28).

#### WHO-5 Well-Being Index

The World Health Organization-5 Well-Being Index (WHO-5 Well-Being Index) was developed to measure the subjective psychological well-being. This five-item scale was revised from the 28-item WHO-10 (29). The respondents answered each item ranging from 0 (none) to 5 (all the time). The final score is calculated by multiplying the raw total score by 4 (30), and a higher score indicates greater psychological well-being. We used the Korean version of this scale in this study (31).

### Statistical Analysis

The demographic variables, clinical characteristics, and rating scales scores are summarized as mean ± standard deviation. The level of significance for the analyses was defined as two-tailed at values of *p* < 0.05. Continuous and categorical variables were analyzed using Student's *t*-test and the chi-squared test, respectively. Correlation analyses of the different variables were conducted using Spearman's correlation analysis since the distribution of the PHQ-9 scale's scores were not within the normal range. To explore the clinical variables influencing the teachers' psychological well-being during the pandemic, we categorized the participants into two groups: high quartile in WHO-5 score (top 25% of participants) and lower three quartiles of WHO-5 score (bottom 75% of participants). A logistic regression analysis was performed to reveal the predicting variables for the teachers' high psychological well-being. In addition, to explore whether teachers' self-efficacy and resilience mediated the effects of stress and anxiety to the pandemic on their depression and psychological well-being, the bootstrap method with 2,000 resamples was implemented. We used SPSS version 21.0 and AMOS version 27 for Windows (IBM Corp., Armonk, NY, USA) to perform the statistical analysis.

## Results

The demographic characteristics of the participants are shown in Table 1. Among the 400 teachers who participated in this study, 140 (35.0%) were working at an elementary school, 130 (32.5%) at a middle school, and 130 (32.5%) at a high school. About two-thirds of the teachers (*n* = 270 [67.5%]) were female, and 177 (44.3%) were single. Their mean age and duration of employment was 38.4 ± 9.1 and 11.8 ± 8.6 years, respectively. They were residents of Seoul (*n* = 78 [19.5%]), Pusan (*n* = 28 [7.0%]), Daegu (*n* = 28 [7.0%]), Daejeon (*n* = 8 [2.0%]), Gwangju (*n* = 10 [2.5%]), Chungcheong Province (*n* = 34 [8.5%]), Jeolla Province (*n* = 31 [7.7%]), Gyeongsang Province (*n* = 49 [12.3%]), Gangwon Province (*n* = 13 [3.3%]), and Jeju Province (*n* = 4 [1.0%]). Seventy-one teachers (17.7%) reported that they had experience caring for children with viral illnesses, 57 (14.2%) were quarantined because of COVID-19 infection, and only one was infected with COVID-19.

**Table 1:** Participants' demographic characteristics (*N* = 400).

**Variable**	**Mean ± SD, *n* (%)**
**School level**	
Elementary	140 (35.0%)
Middle	130 (32.5%)
High	130 (32.5%)
**Sex (female)**	270 (67.5%)
**Age, years**	38.4 ± 9.1
20–29	75 (18.8%)
30–39	161 (40.3%)
40–49	112 (28.0%)
50–59	42 (10.5%)
60–69	10 (2.5%)
**Marital status**	
Single	177 (44.3%)
Married	223 (55.7%)
**Years of employment**	11.8 ± 8.6
**COVID-19 questions**	
Did you care for children who have had viral illnesses? (Yes)	71 (17.7%)
Did you experience being quarantined due to infection with COVID-19? (Yes)	57 (14.2%)
Did you experience being infected with COVID-19? (Yes)	1 (0.3%)
**Psychiatric history**	
Did you experience or treat depression, anxiety, or insomnia? (Yes)	57 (14.2%)
Presently, do you think you are depressed or anxious, or do you need help for your mood state? (Yes)	44 (11.0%)
**Rating scales**	
Stress and Anxiety to Viral Epidemics-9 items	23.6 ± 6.2
Patient Health Questionnaire-9 item	7.6 ± 5.9
Brief Resilience Scale	18.3 ± 5.1
Teacher-Efficacy Scale	92.5 ± 13.5
WHO-5 Well-Being Index	39.5 ± 20.0

*WHO, World Health Organization*.

The Spearman correlation coefficients for participants' years of employment and rating scale scores are shown in Table 2. Years of employment was significantly correlated with teachers' self-efficacy scale score (rho = 0.18, *p* < 0.01). The SAVE-9 scale score was significantly associated with higher PHQ-9 (rho = 0.43, *p* < 0.01), lower WHO-5 (rho = −0.21, *p* < 0.01), and BRS (rho = −0.22, *p* < 0.01) scores. The PHQ-9 score was significantly correlated with a lower WHO-5 score (rho = −0.38, *p* < 0.01), BRS score (rho = −0.53, *p* < 0.01), and teachers' self-efficacy (rho = −0.19, *p* < 0.01). A high WHO-5 score was significantly correlated with high BRS (rho = 0.49, *p* < 0.01) and self-efficacy (rho = 0.25, *p* < 0.01) scores. A higher BRS score was significantly correlated with higher self-efficacy (rho = 0.44, *p* < 0.01).

**Table 2:** Spearman's correlation coefficients of each variable for all participants (*N* = 400).

**Variable**	**Years**	**SAVE-9**	**PHQ-9**	**WHO-5**	**BRS**	**Self-**
	**employment**					**efficacy**
Years of employment	1.000					
SAVE-9	−0.06	1.000				
PHQ-9	−0.03	0.43^**^	1.000			
WHO-5	−0.04	−0.21^**^	−0.38^**^	1.000		
BRS	0.07	−0.22^**^	−0.53^**^	0.49^**^	1.000	
Self-efficacy	0.18^**^	−0.02	−0.19^**^	0.25^**^	0.44^**^	1.000

*BRS, Brief Resilience Scale; PHQ-9, Patient Health Questionnaire-9 items; SAVE-9, Stress and Anxiety to Viral Epidemics-9 items; WHO-5, World Health Organization-5 Well-Being Index; Efficacy, Teacher-Efficacy Scale*.

** *p < 0.01 and*

* *p < 0.05*.

A comparative analysis between participants with high (top 25%) or low (bottom 75%) subjective well-being scores is shown in Table 3. Between the two groups, the proportion of teachers who reported to have current psychiatric symptoms was higher, SAVE-9 and PHQ-9 scores were higher, and BRS and Teacher-Efficacy scale scores were lower in the low subjective well-being group than the high subjective well-being group.

**Table 3:** Comparison of demographic variables and rating scale scores between the low and high subjective well-being groups (*N* = 400).

**Variable**	**WHO-5 top 25%**	**WHO-5 bottom 75%**	***P*-value**
	**(*n* = 103)**	**(*n* = 297)**	
School (elementary, middle, high school)	39.8%, 32.0%, 28.2%	33.3%, 32.7%, 34.0%	0.421
Sex (female)	66 (64.1%)	204 (68.7%)	0.395
Age	38.6 ± 9.7	38.3 ± 8.9	0.825
Years of employment	11.9 ± 9.3	11.7 ± 8.4	0.802
Marital status (single)	45 (43.7%)	132 (44.4%)	0.494
**COVID-19 questions**			
Did you care for children who have had viral illness? (Yes)	25 (24.3%)	46 (15.8%)	0.07
Did you experience being quarantined due to infection with COVID-19? (Yes)	17 (16.5%)	40 (13.5%)	0.447
Did you experience or treat depression, anxiety, or insomnia? (Yes)	11 (10.7%)	46 (15.5%)	0.229
Now, do you think you are depressed or anxious, or do you need help for your mood state? (Yes)	5 (4.9%)	39 (13.1%)	0.021
**Rating scales**			
Stress and Anxiety to Viral Epidemics-9 items	21.6 ± 7.1	24.3 ± 5.7	<0.001
Patient Health Questionnaire-9 items	4.8 ± 5.7	8.6 ± 5.6	<0.001
Brief Resilience Scale	21.8 ± 4.7	17.1 ± 4.7	<0.001
Teacher-Efficacy Scale	99.2 ± 14.0	90.2 ± 12.5	<0.001

*COVID, coronavirus disease; WHO, World Health Organization*.

Logistic regression analysis revealed that high psychological well-being was predicted by lower stress and anxiety to the viral epidemic (adjusted odds ratio [aOR] = 0.95; 95% confidence interval [CI], 0.91–0.99), high self-efficacy (aOR = 1.03; 95% CI, 1.01–1.06), and high resilience (aOR = 1.18; 95% CI, 1.10–1.27) (Table 4).

**Table 4:** Logistic regression analysis for predicting high subjective well-being (WHO-5 top 25%) of teachers during the COVID-19 pandemic.

**Variable**	**cOR**	**(95% CI)**	***p***	**aOR**	**(95% CI)**	***p***
Age	1.002	(0.98–1.03)	0.891	1.01	(0.95–1.08)	0.760
Sex	1.24	(0.77–1.99)	0.375	1.10	(0.63–1.95)	0.736
**Level of school served**						
Elementary						
Middle	0.82	(0.48–1.41)	0.473	0.94	(0.49–1.77)	0.838
High	0.69	(0.40–1.20)	0.192	0.54	(0.28–1.05)	0.068
Years of employment	1.003	(0.98–1.03)	0.801	0.98	(0.92–1.05)	0.652
**Rating scales**						
PHQ-9	0.87	(0.83–092)	<0.001	0.97	(0.91–1.03)	0.289
SAVE-9	0.93	(0.90–0.97)	<0.001	**0.95**	**(0.91–0.99)**	**0.032**
Efficacy	1.06	(1.04–1.08)	<0.001	**1.03**	**(1.01–1.06)**	**0.006**
BRS	1.25	(1.18–1.32)	<0.001	**1.18**	**(1.10–1.27)**	**<0.001**

*aOR, adjusted odds ratio; BRS, Brief Resilience Scale; CI, confidence interval; cOR, crude odds ratio; Efficacy, Teacher-Efficacy Scale; PHQ-9, Patient Health Questionnaire-9 items; SAVE-9, Stress and Anxiety to Viral Epidemics-9 items; WHO-5, World Health Organization-5 Well-Being Index*.

**Figure 1:**
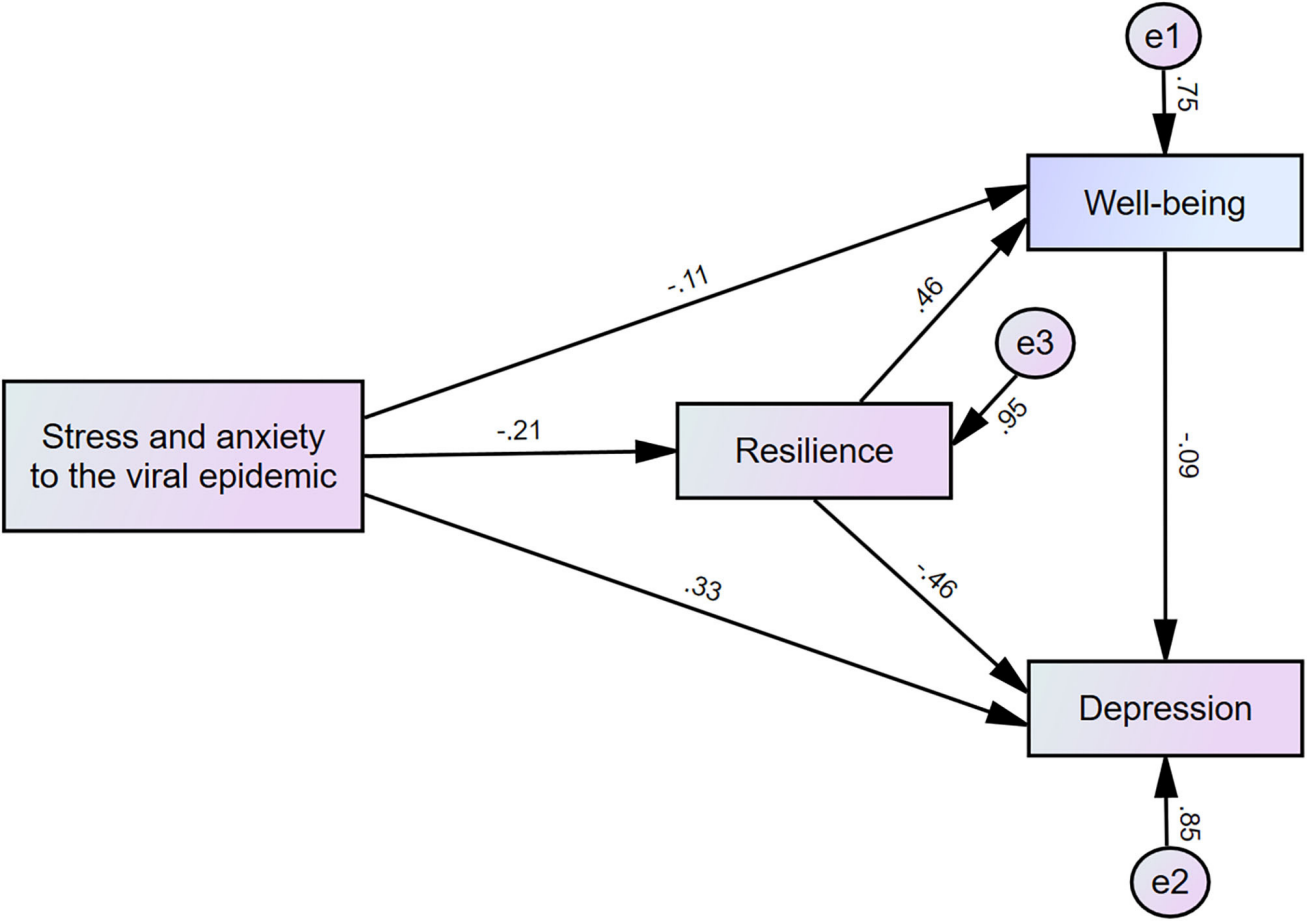
Mediation model showing the pathway from the effect of stress and anxiety to the viral epidemic (independent variables) on psychological well-being or depression (outcome) through resilience (mediator).

A mediation analysis showed that the complete pathway from stress and anxiety to the viral epidemic (independent variable) through resilience (mediator) to teacher psychological well-being (dependent variable) was significant (*z* = −4.05, *p* < 0.001, Table 5A). The pathway from stress and anxiety to the viral epidemic (independent variable) through resilience (mediator) to depression (dependent variable) was significant (*z* = 9.72, *p* < 0.001). This indicates that teacher resilience partially mediates the effects of stress and anxiety on their psychological well-being or depression (Figure 1). However, the pathway from stress and anxiety to the viral epidemic through teacher self-efficacy to psychological well-being or depression was not significant (Table 5B).

**Table 5:** Mediating effect of resilience or self-efficacy on the association between stress and anxiety to the viral epidemic and depression or psychological well-being.

**Effect**	**Standardized estimator**	**S.E**.	***Z*-value**	***P***	**95% CI**
**(A) Mediating effect of resilience**					
**Direct effect**					
SAVE-9 → WHO-5	−0.11	0.04	−2.55	0.011	−0.20 ~−0.03
SAVE-9 → PHQ-9	0.33	0.04	8.53	<0.001	0.26 ~ 0.41
**Indirect effect**					
SAVE-9 → BRS → WHO-5	−0.10	0.02	−4.11	<0.001	−0.15 ~−0.05
SAVE-9 → BRS → PHQ-9	0.10	0.02	4.07	<0.001	0.05 ~ 0.15
**Total effect**					
SAVE-9 → WHO-5	−0.21	0.05	−4.05	<0.001	−0.31 ~−0.11
SAVE-9 → PHQ-9	0.43	0.04	9.72	<0.001	0.34 ~ 0.52
**(B) Mediating effect of self-efficacy**					
**Direct effect**					
SAVE-9 → WHO-5	−0.21	0.05	−4.13	<0.001	−0.31 ~−0.11
SAVE-9 → PHQ-9	0.43	0.04	9.95	<0.001	0.34 ~ 0.51
**Indirect effect**					
SAVE-9 → Efficacy → WHO-5	−0.003	0.01	−0.21	0.83	−0.03 ~ 0.02
SAVE-9 → Efficacy → PHQ-9	0.002	0.01	0.21	0.83	−0.02 ~ 0.02
**Total effect**					
SAVE-9 → WHO-5	−0.21	0.05	−4.05	<0.001	−0.31 ~−0.11
SAVE-9 → PHQ-9	0.43	0.04	9.72	<0.001	0.34 ~ 0.52

*BRS, Brief Resilience Scale; CI, confidence interval; Efficacy, Teacher-Efficacy Scale; PHQ-9, Patient Health Questionnaire-9 items; SAVE-9, Stress and Anxiety to Viral Epidemics-9 items; S.E., standard error; WHO-5, World Health Organization-5 Well-Being Index*.

## Discussion

In this study, we observed that schoolteachers' psychological well-being may be influenced by lower stress and anxiety levels due to the pandemic, higher self-efficacy, and higher resilience. We also observed that a teacher's resilience mediates the influence of stress and anxiety due to the COVID-19 pandemic on their depression or psychological well-being. However, we also observed that teachers' self-efficacy does not mediate this association.

In this study, we used the schoolteachers' version of the newly developed SAVE-9 scale (23) to assess schoolteachers' stress and anxiety responses to the viral epidemic. We could not use the well-known anxiety measures such as Generalized Anxiety Disorder-7 items or State and Trait Anxiety Inventory to observe the COVID-19–specific anxiety response of schoolteachers in this pandemic. Therefore, viral epidemic-specific anxiety measures should be applied. The results from the logistic regression analysis in this study revealed that the low level of stress and anxiety to the viral epidemic was a significant variable for the high level of psychological well-being of schoolteachers. In the COVID-19 era, changes in the school environment have led to higher teacher stress and burnout rates. According to a study conducted in the United States in October 2020, several stressors related to the pandemic play a major role in accelerating teachers' burnout, which include COVID-19-related anxiety as well as anxiety about teaching demands, parent communication, and administrative support (32).

Technical difficulties in distance teaching (33) and responsibility for health measures adopted in schools (34) have also increased stress for teachers. Previous studies revealed that even before the COVID pandemic, teaching was among the most stressful occupations worldwide, with various sources of stress contributing to a lower than average QOL, including personal life stress and work-life stress (35, 36). A longitudinal study in Chile showed that the pandemic significantly worsened teachers' QOL (37). This result cannot be overlooked because the low QOL of teachers may lead to serious consequences for the teachers themselves as well as the students in their care. This may worsen the general school environment. Thus, elucidating the factors that may influence teachers' psychological well-being in the COVID era is of utmost importance. To our knowledge, this study is the first to examine various factors that influence teachers' psychological well-being during the present pandemic.

In this study, the logistic regression analysis showed that teachers' resilience and self-efficacy are meaningful factors in deciding their psychological well-being during the pandemic. Notably, high resilience can increase teachers' job satisfaction, commitment, efficacy, motivation, engagement, and well-being (38). A prospective controlled trial in Israel showed that resilience was positively correlated with psychological well-being, life satisfaction, and positive affect and negative correlations with emotional exhaustion during the pandemic (39). Furthermore, according to a descriptive analytical study conducted in primary, secondary, and high schools of Shahrekord city, Iran, teachers' QOL was significantly positively correlated with their professional self-efficacy (40). Baloran et al. (41) identified a positive correlation between crisis self-efficacy and the work commitment of teachers in the COVID situation. Throughout our study, we observed that resilience and self-efficacy were factors contributing to teachers' higher psychological well-being, even during the pandemic.

Through the mediation analysis, resilience was more adaptive to the mediation model than self-efficacy. The fact that resilience has a mediating effect indicates that stress and anxiety to COVID-19 decreases resilience, which results in increased depression or worsened psychological well-being. According to a review of past studies on teacher resilience, negative self-belief or confidence is the most commonly described risk factor for resilience (12). In our study, several SAVE-9 items were correlated with negative self-belief or confidence, such as “Are you afraid your health will worsen because of the virus?”, “Are you worried that you might get infected?”, “Are you worried that others might avoid you even after the infection risk has been minimized?”, and “Do you think that your colleagues would have more work to do due to your absence from a possible quarantine and might blame you?”. Therefore, it can be assumed that participants with more stress and anxiety to COVID-19 were likely to have decreased resilience levels. It is difficult to directly compare our results to those of previous studies because no previous studies examined the mediating effect of resilience on the relationship between stress or anxiety to COVID-19 and psychological well-being or depression. However, our study findings are in line with previous studies showing the mediating effect of resilience on the relationship between emotional labor and happiness of clinical nurses (42) or the relationship between academic burnout and the psychological well-being of medical students (43).

In contrast, self-efficacy had no mediating effect in our study. Several previous studies showed the mediating effect of self-efficacy. In a study of 282 Korean undergraduate students, a mediation effect of self-efficacy was found with respect to the relationship between perceived stress and life satisfaction (44). Another study of 207 Chinese students in Hong Kong showed the mediating role of self-efficacy on the effects of adjustment problems on psychological distress (45). The mediating role of teacher self-efficacy was also discussed in a study in China, which found that teacher self-efficacy partially mediated work stress and prevented burnout (46). Nevertheless, in our study, even though teacher self-efficacy was an important factor in deciding their psychological well-being during the pandemic, it did not act as a mediator between stress or anxiety to COVID-19 and their psychological well-being. Factors generally known to influence teacher self-efficacy are as follows: expectations of students and parents, workload, and working conditions such as pay, work other than teaching, and social recognition. Specifically in the pandemic era, there has been a sudden change in teachers' workload and working conditions, such as engaging in online teaching and taking preventive measures against the virus, such as having to supervise students' mask wearing (33). However, our study was conducted during March 4–15, 2021, already about a year after the outbreak first began in South Korea. Most of the teachers became accustomed to the new teaching environment. Therefore, it is possible that COVID-19–specific factors did not play a major role in determining teacher efficacy during our study. Additionally, when we consider the participants' demographic characteristics, 59% of the participants were younger than 40 years, while 87% were younger than 50 years. Because younger teachers generally adapt more easily to online teaching than older teachers, this may be another explanation why teacher efficacy was not significantly influenced by COVID-19–related factors in our study. Few previous studies examined factors contributing to teacher efficacy during the pandemic, but one that included 130 high school teachers in Korea concluded that depression and anxiety did not significantly influence their efficacy during the pandemic (47). Our study findings partially support this result.

The present study has a few limitations. First, its results cannot answer whether resilience or self-efficacy contribute to schoolteacher stress or psychological well-being regardless of the COVID-19 pandemic. We knew that the resilience, but not self-efficacy, of schoolteachers mediated the influence of stress and anxiety due to the COVID-19 pandemic on their depression or psychological well-being. However, we could not compare the relationship between periods prior to the pandemic and after the pandemic. Second, this study was conducted via an anonymous online survey. The relatively small sample of patients selected from a national area can reduce the statistical power of this study. We conducted this study via online surveys rather than face-to-face interviews, to prevent the risk of a viral outbreak. In COVID-19 pandemic chaos, it is difficult to gather enough participants from all national areas. Third, this survey was conducted in 2021; thus, its participants could have adapted to the COVID-19 school environment by that point. They had experience taking care of students, creating online education materials, and educating students virtually. Their prior experience might influence their psychological status. Fourth, the results of this study may be biased by the small sample size and limited area, South Korea; thus, it may not reflect the real school environment in other nations. Also, samples of schoolteachers from diverse school levels (elementary, middle, or high) might make the interpretation of results difficult as learners have different requirements at different levels; hence, the challenges faced by the teachers differ. However, the participants were enrolled from various regions across South Korea, a strength of this study. Furthermore, there may be selection bias due to teachers being tech-literate and able to access an online survey easily and participate in this study, and 10% of low response rate (400 participants among 4,000 schoolteachers to whom the company sent an email for the participation) might have influenced its results.

In conclusion, we observed that schoolteachers' psychological well-being or depression were influenced by their stress and anxiety due to the viral epidemic in this pandemic era, and their resilience mediated the influence of stress and anxiety due to the COVID-19 pandemic on their depression or psychological well-being. This conclusion may contribute to the development of a psychological support system for teachers working in schools in this pandemic.

## Data Availability Statement

The raw data supporting the conclusions of this article will be made available by the authors, without undue reservation.

## Ethics Statement

The studies involving human participants were reviewed and approved by the participants responded to the survey voluntarily. This study protocol was approved by the Institutional Review Board of Asan Medical Center (2021-0098), and written informed consent was waived. Written informed consent for participation was not required for this study in accordance with the national legislation and the institutional requirements.

## Author Contributions

SC and SS: conceptualization. SC, JL, I-KC, and KK: data curation. SC, SL, JL, and SY: formal analysis. SY and SL: investigations. SC, SY, SS, and SL: methodology. SC: project administration. SS: resources. JL, KK, and I-KC: visualization. I-KC and SC: writing—original draft. All authors writing—review and editing.

## Funding

This work was supported under the Framework of International Cooperation Program managed by the National Research Foundation of Korea (FY2020K2A9A1A01094956).

## Conflict of Interest

The authors declare that the research was conducted in the absence of any commercial or financial relationships that could be construed as a potential conflict of interest.

## Publisher's Note

All claims expressed in this article are solely those of the authors and do not necessarily represent those of their affiliated organizations, or those of the publisher, the editors and the reviewers. Any product that may be evaluated in this article, or claim that may be made by its manufacturer, is not guaranteed or endorsed by the publisher.

## Footnotes

^1^Available online at: http://ncov.mohw.go.kr/en/ (accessed March 25, 2021).

^2^Available online at: https://www.see-partnerships.com/uploads/1/3/2/8/132824390/va_covid_teacher_depression.pdf (accessed September 02, 2021).

^3^Available online at: https://www.moe.go.kr/boardCnts/view.do?boardID=294&boardSeq=81682&lev=0&searchType=null&statusYN=W&page=1&s=moe&m=020402&opType=N (accessed September 02, 2021).
